# An Account of Diseases in an Eastern District of London from May 20, to June 20, 1809

**Published:** 1809-07

**Authors:** 


*An Account of Diseases in an Eastern District of London from May 20, to June 20, 1809.*

| | |
|---|---|
| ACUTE DISEASES. | |
| Pneumonia - - - - | 3 |
| Peripneumonia Notha | 2 |
| Dysenteria - - - - - | 1 |
| Rheumatismus Acutus | 4 |
| CHRONIC DISEASES. | |
| Tussis - - - - - - - | 17 |
| Dyspnœa - - - - - - | 5 |
| Tussis cum Dyspnœa - | 12 |
| Hæmoptysis - - - - - | 3 |
| Phthisis - - - - - - | 2 |
| Pleurodyne - - - - - | 4 |
| Hydrothorax - - - - - | 5 |
| Hepatitis Chronica - - | 3 |
| Enterodynia - - - - - | 2 |
| Diarrhœa - - - - - | 4 |
| Hæmorrhois - - - - - | 1 |
| Menorrhagia - - - - - | 5 |
| Leucorrhœa - - - - | 7 |
| Rheumatismus Chronicus | 14 |
| PUERPERAL DISEASES. | |
| Menorrhagia Lochialis | 3 |
| Mastodynia - - - - | 4 |
| Rhagas Papillæ - - - | 3 |
| Dolores post Partum - | 6 |
| INFANTILE DISEASES. | |
| Pertussis - - - - - - | 3 |
| Aphthæ - - - - - - | 3 |
| Diarrhœa - - - - - - | 4 |
| Dentitio - - - - - - | 2 |
| Vermes - - - - - - - | 2 |

Notwithstanding the season is so far advanced, and we have for some time entered on the Summer Quarter, the state of the weather, and the temperature of the air, have been such as might be expected in the months of March and April. During the prevalence of the high winds, which have been blowing in all directions, and particularly in consequence of those which have proceeded in a North and North Eastern direction, much mischief has been done, both to the vegetable and the animal world. The trees of the forest have suffered much in their foliage, and the fruit trees have been despoiled of their blossoms. The human frame, as might be expected, has partaken of the injury; catarrhal complaints have been very frequent, and some severe affections of the chest have been experienced. Our list of diseases, therefore, bears a near resemblance to those which we have presented for some months past. Until within a few days, such has been the vicissitude of temperature, that different pneumonic symptoms have, in some instances, been almost as severe as in the winter months, and seem to threaten consequences similar to those referred to in preceding Reports.

